# Wound Healing Modulation in Glaucoma Filtration Surgery–Conventional Practices and New Perspectives: The Role of Antifibrotic Agents (Part I)

**DOI:** 10.5005/jp-journals-10008-1159

**Published:** 2014-06-12

**Authors:** Jennifer C Fan Gaskin, Dan Q Nguyen, Ghee Soon Ang, Jeremy O'Connor, Jonathan G Crowston

**Affiliations:** Glaucoma Fellow, Glaucoma Investigation and Research Unit, Centre for Eye Research, University of Melbourne, Melbourne, Australia; Consultant, Department of Ophthalmology, Mid Cheshire Hospitals, NHS Foundation Trust, Cheshire; Institute for Science and Technology in Medicine, Keele University, Keele, Staffordshire, UK; Consultant, Glaucoma Investigation and Research Unit, Centre for Eye Research, University of Melbourne, Melbourne, Australia; Consultant, Glaucoma Investigation and Research Unit, University Hospital Limerick, Ireland; Pofessor, Glaucoma Investigation and Research Unit, Centre for Eye Research, University of Melbourne, Melbourne, Australia

**Keywords:** Glaucoma, Trabeculectomy, Filtration surgery, Antimetabolites, Wound healing modulation, Scarring.

## Abstract

Glaucoma filtration surgery is regularly performed for the treatment of glaucoma and trabeculectomy is often regarded as the ‘gold standard' glaucoma operation. The biggest risk of failure of the operation is bleb scarring. The advent of anti-fibrotic agents, such as mitomycin C (MMC) and 5-fluorouracil (5FU) has vastly prolonged the longevity of the bleb, but concerns remain regarding the potential increase in postoperative complications. More selective therapeutic targets have therefore been explored. One of these is vascular endothelial growth factor (VEGF) inhibition. Vascular endothelial growth factor inhi bition has a role not only in sub conjunctival angiogenesis inhi bition but also it has direct anti-fibrotic properties. Newer phar macological compounds and materials have also been developed in recent years in attempt to modulate the wound healing in different ways after glaucoma surgery. These include physical barriers to scarring and vehicles for sustained release of pharmacological agents, and early promising results have been demonstrated. This two-part review will provide a discussion of the application of anti-fibrotic agents in glaucoma filtration surgery and evaluate the newer agents that have been developed.

**How to cite this article:** Fan Gaskin JC, Nguyen DQ, Ang GS, O'Connor J, Crowston JG. Wound Healing Modulation in Glaucoma Filtration Surgery–Conventional Practices and New Pers pectives: The Role of Antifibrotic Agents (Part I). J Curr Glaucoma Pract 2014;8(2):37-45.

## INTRODUCTION

It has long been recognised that fibrosis of the filtration site is one of the main reasons for primary failure of glaucoma filtering surgery. Since the initial description of the use of mitomycin C (MMC) as an adjunct to trabeculectomy in 1983 by Chen,^1^ and echoed a year later by Gressel with 5-fluorouracil (5FU),^2^ the filtering bleb has seen greater success both in its efficacy and longevity. These two agents have been widely adopted in an off-label capacity to combat tissue fibrosis following surgery. However, the use of anti-fibrotic agents is not without its risks and limitations. Other more alternative approaches to wound modulation in glaucoma surgery have therefore been explored in recent years, including anti-vascular endothelial growth factor (anti-VEGF). This two-part series will review the current practice in wound healing modulation in glaucoma surgery and explore the newer options on the horizon. Part one will provide an overview of the current use of MMC and 5FU as anti-fibrotic agents in glaucoma filtration surgery, and part two will discuss the use of anti-VEGF as well as other more novel agents/materials being developed.

## REVIEW

### Rationale for Wound Healing Modulation

A trabeculectomy bleb, like any other tissue, undergoes different phases of postoperative wound healing.^3^ The first phase is an immediate Inflammatory response that occurs in the initial postoperative days. This phase is characterized by recruitment of in fam matory cells, cytokines and growth factors, triggering the onset of the second phase, proliferation and tissue reparation, which commences in the early postoperative days and can continue into the second or third postoperative months. The second phase comprises activation, migration and proliferation of episcleral fibro-blasts, angio genesis and the formation of collagen bundles. The third and final phase involves remodelling and final healing of the wound, when contraction of the collagen fibers and formation of scar tissue occurs, ultimately resulting in reduction of aqueousdrainage and sub conjunctival absorp-tion. While healing under the scleral fap may play a role in some cases, it is the fibroblasts in Tenon's capsule that are the main effector cells in the initia tion and mediation of trabeculectomy wou nd healing and fibrotic scar for mation.^4^

The purpose of wound healing modulation is to moderate the healing process through pharmacological inter vent ion. It involves the delicate balance of optimal conjunctival healing with the suppression of scar formation in the filtering bleb. In the most rudimentary form, it comprises the application of topical corticosteroid eye drops in the postoperative period. A more potent form of wound healing modulation requires the subtenons and/or sub-scleral fap application of an antimet abolite, such as MMC or 5FU intra operatively; 5FU can also be applied as repeated subconjunctival injections postoperatively.

### Mechanism of Action and Administration of Mitomycin C

Mitomycin C is an alkaloid produced by the bacterium *Streptomyces caespitosus*. The direct cytotoxic effect of MMC occurs through inhibition of DNA-dependent RNA-synthesis.^5^ Induction of apoptosis has been demonstrated in cultured human Tenon's fibroblasts following application of MMC (concentration range: 0.1-1.0 mg/ml) for 5 minutes.^6^ The apoptotic process begins 48 hours after administration of MMC *in vitro* and the amount of apoptosis induced is dose-dependent.^6^ Mitomycin C has also been observed to increase the expression of apoptosis genes in human Tenon's fibroblasts.^7^ Mitomycin C contains quinine, urethane and aziridine, all of which have anti-tumor properties. It also has potent anti-angiogenic properties.^8^ The effect of intraoperative application of MMC has been demonstrated to be localized to the area it is exposed to.^8^ Furthermore, the intracellular activation of MMC to the active reduced form may occur more efficiently in ocular fibroblasts than in fibroblasts from other parts of the body.^9^ Therefore, the many properties of MMC are utilized during trabeculectomy to modulate wound healing in the subconjunctival space as well as Tenon's capsule.^10^

Mitomycin C is usually soaked into cellulose sponges and applied bet ween the conjunctiva-Te non's capsule and the sclera before scleral fap dissect ion;^11,12^ following scleral fapdissection with application under the scleralfap;^4,13^ or both. It has also been reported to be injected subconjunctivally rather than applied with cellulosesponges .^14^ El Sayyad et al demonst rated significantly lower IOP cont rol in eye streated with MMC application both under the scleral fap and the conjunctiva during trabeculectomy compared to under the scleral fap or conjunctival fap alone at 12 months follow-up.^15^ Similarly, Prata et al also found greater likelihood of achieving IOP ≤ 21 mm Hg in eyes treated with MMC under the dissected scleral fap and conjunctiva compared to on the episclera alone, without any significant difference in rates of complication.^16^ However, inafascinating study, Gandolf etal demonstrated that IOP could be reduced merely by injecting MMC subcon junc tivally without an accompanying trabeculectomy in eyes already blinded by glaucoma. This IOP-lowering effect was detectable even after 60 days of follow-up.^17^ Asnoalteration was made to the ocular outfow system during this study, the authors postulated that the reduction in IOP in fact resulted from the toxic effects of MMC diffusing into the ciliary body. This idea has been supported by earlieranimalmodels.^18,19^ Increasing concentrations of MMC in the deeper scleral layers with increasing MMC diffusion time has also been demonstrated by Georgopoulos et al,^20^ who observed that after 1 minute application of 0.2 mg/ml of MMC to donor human sclera, there was no difference in MMC concentration across the scler a after 30 minuted iffusion time. Si nce MMC continues to diffuse into the sclera following its application in human trabeculectomy, it is conceivable that MMC may damage the ciliary body and therefore result in a hypotonic effect.

The concentration of MMC used in glaucoma filtration surgery from 0.1 to 0.5 mg/ml in a dilution with balanced salt solution. The concentration and duration of application are determined arbitrarily by the surgeon, depending on their assessment of the patient and the risk of surgical failure. Vass et al.^21^ assessed the intrascleral concen tration of MMC in different layers following episcleral application of varying concentrations of MMC (0.01 mg /ml, 0.1 mg /ml, 0.2 mg/ml) in human donor sclera for 1 minute and identified statistically significant differences in the intrascleral concentrations of MMC with varying concentrations of applied MMC. There was a log-linear decrease in MMC concentration with increasing depth of sclera, and this was observed with all three MMC concent rations. Howeve r, thus far, the majority of clinical studies have failed to demonstrate a significant difference in final IOP based on variations in MMC concentration or exposure time.^22-27^ Only one study of 300 eyes has identified a possible dose-response relationship between the concentration and duration of exposure to MMC although the differences were not statistically significant.^28^

Immediately following the application of MMC, the treated area and especially the surrounding conjunctiva is typically rinsed with 20 to 50 ml of nor mal saline to prevent toxicity to the surrounding tissues. Vass et al.^29^ assessed the effect of saline irrigation on MMC treated human donor sclera and found decreased MMC concentration in the superficial layers of the sclera after irrigation compared to no irrigation. The concentration of MMC decreased in these layers with increa sing volume of irrigating saline (40-200 ml). However, in the deeper scleral layers, irrigation made no difference to MMC concentration. It is worth noting that this study was conducted on scleral quadrants mounted on watertight shields that may have infuenced the effect of irrigation on the deeper layers. It is possible that *in vivo*, irrigation does actually make a difference to the MMC concentration in the deep scleral layers.

### Clinical Indications for MMc-augmented Trabeculectomy

Mitomycin C is inexpensive and simple to use as a one-off intraoperative application. The main reason that MMC has been so readily adopted by ophthalmologists is its efficacy in modulating post-trabeculectomy conjunctival scarring. A recent survey of United Kingdom (UK) glaucoma units revealed anti fibrotic use in 93% of its primary trabecu-lectomies with 63% being MMC.^30^ In contrast, in the 1995 UK National Survey of Trabeculectomy, only 6.4% of trabeculectomies were performed with an adjunctive anti-fibrotic.^31^ A similar survey of the American Glaucoma Society found that MMC use in primary trabeculectomies increased from 45% in 1996 to 68% in 2002.^32^

Its efficacy is best illustrated by its use in trabeculectomy for primary open-angle glaucoma (POAG). Reibaldi et al^33^ con-duc ted a randomized clinical trial comparing 0.2 mg/ml MMC with balanced salt solution (BSS) applied intraoperatively on the episclera during trabeculectomy in 114 eyes of 114 patients. A significantly higher proportion of patients achieved IOP ≤ 18 mm Hg without medication (71% *vs* 51%, p = 0.027) and with IOP ≤ 14 mm Hg (57% *vs* 32%, p = 0.015) in the MMC-treated group compared to the BSS-treated group over a 9-year follow-up period. Corres pondingly, a lower proportion in the MMC group developed visual field progression (22% *vs* 47%, p = 0.009) or required further glaucoma surgery (9% *vs* 26%, p = 0.040) compared to the BSS group. Similar results have been demonstrated in other studies.^34-36^ In young adults (< 40 years), unaugmented trabeculectomy is less likely to be successful due to a more vigorous wound healing response and potential for scarring,^37,38^ but survival of the operation and postoperative IOP control can be improved with adjunctive MMC during su rger y.^39^

In normal tension glaucoma (NTG), although filtering surgery may achieve a higher percentage of IOP reduction compared to topical medication, there is an increased rate of sight-threatening complications, such as late bleb leak and late hypotony when adjunctive MMC is administered.^40^ Alt hough the aut hors have demonst rated an overall decrea se in the risk of visual field deterioration with increasing per-cen tage fall in IOP postoperatively from baseline, patients who received MMC had the worst visual field sur vival despite having the greatest fall in IOP compared to eyes with higher postoperative IOPs following non-augmented trabeculectomy or trabeculectomy with 5FU.^41^ The authors postulated that the higher rate of postoperative complications, including visual acuity loss and hypotony, in the MMC group may haveled to consequences that account for the greater rate of visual field progression.^41^

Trabeculectomy with MMC has been shown to be a good solution for congenital glaucomas unresponsive to conven tional surgical treatment, such as goniotomy or trabe cu lotomy, with a success rate of maintaining adequate IOP control of 59 to 95%.^42-44^ Although the number of patients studied was small (19 eyes of 13 patients), Mandal et al. demonstrated a 95% complete success rate (IOP < 21 mm Hg at slit lamp or < 16 mm Hg with patients under general anesthesia, no progression of disk cupping and no enlargement of corneal diameter in the absence of topical glaucoma medication) with maintained visual acuity at up to 20 months following MMC-augmented trabeculectomy for primary congenital glaucoma.^42^ There were no cases of bleb-related infections in this series. Al-Hazmi et al. published a larger series of 150 children who underwent trabeculectomy with MMC and found 62% maintained IOP control of < 21 mm Hg after at least 1-year follow-up.^44^ They did not detect any difference between 0.2 mg/ml and 0.4 mg/ml concentration of MMC with 0.4 mg/ml. The rate of bleb-related infection rate in this series was 1.2%. In a retrospective series of 29 patients with childhood glaucoma, Sidoti et al. observed a success rate of IOP cont rol of 59% at 36 months, with 17% bleb-related infection rate at a mean follow-up of 28 months. The authors acknowledged that the relatively high dose of MMC exposure in their series (0.5 mg/ml applied for an average time of 3.8 minutes) may be related to the higher infection rate and lower IOP maintenance rate. Of all the childhood glaucomas, aphakic glaucoma has been repeatedly highlighted to have a poor success rate following MMC-augmented trabeculectomy, with a success rate of maintaining IOP of ≤ 21 mm Hg between 0 and 37%,^45,46^ and a risk ratio of failure of 2.7.^47^ Age of less than 1 year at time of surgery also carries a high risk of failure.^47^

Trabeculectomy with MMC has an overall lower success rate in secondary glaucomas compared to primary glau comas. In post-traumatic angle recession glaucoma, the cumu lative probability of success was 85% at 1 year, decreasing to 66% at 3 years.^48,49^ Although uveitis is generally considered to be a risk factor for failure of glaucoma filtering surgery,^50^ Kaburaki et al. observed that if aug mented trabeculectomy is performed in inactive uveitic eyes without prior intraocular surgery, it has a similar probability of success as patients with POAG.^51^ However, the presence of postoperative Inflammation significantly reduced the likelihood of success.^51^

In neovascular glaucoma (NVG), obstruction of the trabe cular meshwork by proliferating fibrovascular tissue can lead to permanent peripheral anterior synechiae, resulting in elevated IOP that is difficult to control. Trabe-cu lectomy with MMC may be a reason able treatment option for poorly controlled NVG once retinal ischemia has been treated, particularly in eyes with good visual potential. Kiuchi et al. demonstrated a cumulative probability of success following MMC-aug mented trabeculectomy of 62% after 2 to 3 years in patients with NVG secondary to diabeticretinopathy.^52^ A si m ilar outcome was published by Takihara et al. in a study of patients with NVG from all causes (52% probability of success at 5 years).^53^ Both studies found an association between previous vitrectomy and increased likelihood of trabe culectomy failure.

Bleb needle revision follow ing failed t rabe cu lect omy has also been reported to have higher success when augmented with MMC.^54-56^ In this set ting, MMC can be applied trans-conjunc tivally after being soaked into a cellulose sponge or as a subconjunctival injection. Corneal endothelial safety has been confirmed through specular microscopic assess ment of the endothelium pre- and post- MMC bleb needling.^54^

In a meta-analysis assessing the role of MMC in non-pene trating glaucoma surgery (NPGS), MMC has been identified to be associated with greater IOP reduction and greater likelihood of complete success compared to the lack of MMC use.^57^ The benefit of MMC is sustained at 36 months.^57^ Intraoperative MMC was not associated with greater risk of complications in NPGS.

There is currently no consensus of MMC usein conjunc-tion with insertion of glaucoma drainage devices, with some authors reporting greater likelihood of surgical success with MMC,^58^ whilst others reporting no significant difference,^59^ or less likelihood of success.^60^

### Mechanism of Action and Administration with 5Fu

5-fluorouracil is a pyrimidine analog which inhibits incorporation of thymidine into DNA, and interferes with RNA and ribosomal RNA synthesis.^61^ It has been utilized in reducing episcleral scar formation in glaucoma filtering surgery as it induces apoptosis of fibroblasts in Tenon's capsule in a time- and concentration-dependent manner.^62^ It is less potent than MMC in that the percentage of apop-totic cell death induced in human Tenon's fibroblasts is less when compared to MMC, even when a high dose 5FU is used (up to 50 mg/ml); this is due to its less direct DNA damage compared to MMC.^6^ Its effects are cycle-specific and mediated primarily on proliferating cells.^6^ Unlike MMC, potent anti-VEGF properties have not been demonstrated.^54,63^

Similar to MMC, 5FU can be applied intraoperatively under the conjunctival and scleral faps with a typical dosage of 25 to 50 mg for 5 minutes.^64-66^ Alternatively, it can be administered as repeated subconjunctival injections in the postoperative period in the of fice setting, with a typical dose per injection of 5 mg in 0.1 to 0.5 ml of saline (5%).^61^ The number and frequency of postoperative injections depend on the clinical profile of the patient and clinical response.^67-70^

### Clinical Indications for 5Fu Augmented Trabeculectomy

The Fluorouracil Filtering Surgery Study Group observed that in patients deemed to be at higher risk of bleb failure, such as those with previous cataract surgery or failed filtration surgery, trabeculectomy is more likely to have long-term success if repeated postoperative subconjunctival 5FU injections are administered.^69^ The 5-year cumulative success rate for eyes with a history of cataract surgery was 48% in the 5FU group and 23% in the control group ( p < 0.0 01). For phakic eyes with a failed filtering procedure, the 5-year cumulative success rate was 47% for the 5FU group *vs* 17% for the control group (p = 0.009). The overall risk of failure of the operation was significantly lower in the 5FU group compared to the control group (51% *vs* 74% , p < 0.001). It must be noted that these investigators utilised an intensive 5FU regimen involving twice-daily 5FU injections for the first 7 postoperative days, and then daily injections from days 8 to 14, which is far more intensive than what normally occurs within the clinical setting.

In very high-risk patients, such as those with neovascular glaucoma, trabeculectomy with adjunctive 5FU may be deemed successful in it ially but still has a likeli hood of early failure. One study reported a median filter survival time of 39 months with a success rate of 28% at 5 years.^71^ Other authors reported 70% failure rate to occur within the first 2 months.^72^ These patients are also more likely to develop postoperative complications and poor outcomes, with 35% of patients losing light perception, and 24% developing phthisis bulbi.^71^

In low-risk patients without previous intraocular surgery, it is unclear whether trabeculectomy with repeated postoperative 5FU injections improves the long-term success of the surgery when compared to trabeculectomy alone. Leyland et al.^73^ conducted a double-blind, randomized controlled trial comparing intraoperative application of 5FU (25 mg/ml for 5 minutes) to sodium chloride and identified no significant difference in IOP control at the 12 months follow-up or final follow-up (up to 52 months) in 40 eyes of 36 patients. Rothman et al. observed a cumulative 5-year success rate (IOP ≤ 21 mm Hg) of 78% in the 5FU group com pared to 62% in the control group (p = 0.02).^74^ The Singapore 5-fluorouracil trial reported a higher proportion of patients in the 5FU group than the placebo group with success of surgery (IOP > 17 mm Hg or < 6 mm Hg) at 3 years (p = 0.0154);^75^ however, at 8 years the 5FU group only demonstrated higher likelihood of success if failure was defined as IOP > 14 mm Hg with or without medications (p = 0.04).^76^

### Comparison Between MMc and 5Fu in Glaucoma Filtration Surgery

Studies comparing the efficacy of MMC *vs* 5FU have shown variable results. In the largest prospective, randomised controlled trial comparing the two agents, Singh et al.^65^ reviewed the results of 108 eyes of 108 glaucoma patients with POAG, pseudoexfoliative, pigmentary, or pri mary angle closure glaucoma that received either trabe culectomy with intraoperative MMC (0.4 mg/ml for 2 minutes) or intraoperative subconjunctival 5FU. Additionally, participating surgeons could also give postoperative 5F U injections if deemed necessary clinically. Their results demonstrated no significant difference in complete or qualified success rates between the two groups, nor were there any differences in the short- and long-term complication rates.

WuDunn et al. conducted a similar study by randomizing patients (115 eyes of 103 subjects) without prior intraocular surgery to intraoperative 5FU or 0.2 mg/ml MMC in trabecu lectomy and their results also showed no significant difference between the two groups in terms of success or complication rates. This was consistent both at 12 months and long-term follow-up.^64,77^

However, two smaller randomized controlled trials repor-ted diffierent results. Singh et al. observed that the two agents were equally safe but MMC was more efficacious in a study of 85 eyes of 85 West African glaucoma patients.^78^ Lamping and Belkin reported the same conclusion in their study of 80 eyes of 74 pseudophakic patients with glaucoma.^79^

Anand and Khan looked at the efficacy of the two agents for bleb needle revision following failed trabeculectomies by retrospectively analyzing the outcomes of 98 eyes of 95 patients who underwent slit-lamp bleb-needling augmented with either MMC or 5FU.^80^ Eyes with MMC-needling had longer bleb survival time compared to 5FU (36 months *vs* 8 months, p = 0.009).^80^ Sixty-one percent of eyes post MMC-needling *vs* 30% of eyes post 5FU needling were able to maintain an IOP of 5 to 16 mm Hg with no glaucoma medications or further procedures after 2 years.^80^

### Complications and Drawbacks of Antimetabolite use

Despite the evidence behind the efficacy of adjunctive antimet abolites, the iruseh as also increased the complication rate of filtration surgery. One of the main complications is late-onset leak (>3 months after surgery) related to bleb ischemia and breakdown of the conjunctiva.^81^ Leaks are problematic because, if left untreated, they can cause vision-threatening hypotony maculopathy, shallowing of the anterior chamber, peripheral anterior synechiae, cataract formation, corneal decompensation, choroidal effusion, suprachoroidal hemorrhage and endophthalmitis. The incidence of late bleb leaks after full-thickness surgery in the pre-antimetabolite era was 3.3%.^82^ This decreased with the introduction of partial thickness, guarded trabeculectomies but has been reported to have increased again with the use of antimetabolites, potentially up to as high as 30% in some series.^11,22,24,69,83,84^

The Moorfield's safer surgery system was developed to reduce the risks of trabeculectomy complications while maintaining its efficacy in lowering IOP.^85^ To reduce bleb-related complications, this system advocates two main points intraoperatively: a fornix-based (rather than a limbus-based) conjunctival incision and a larger antimetabolite treatment area. The underlying principle is to eliminate the risk factors that lead to small, avascular, cystic blebs that are more likely to leak, including limbus-based incisions,^86^ and small antimetabolite treatment areas.^87^

Mitomycin C-trabeculectomy is associated with prolonged hypotony (IOP < 6 mm Hg for longer than 14 d ays)^88,89^ and has a reported incidence of approximately 2.9%.^90^ If hypotony maculopathy develops, visual function will be significantly reduced and may not recover even when the hypotony is reversed.^91^ Hypotony is more commonly seen with higher concentration and longer exposure of MMC, presumably due to increased penetration of MMC into the ocular tissues.^27^ Myopia ^91,92^ and youngera ge ^88,91,92^ are other risk factors for prolonged postoperative hypotony.

Of all the bleb-related complications, perhaps the most devastating of all is bleb-related endophthalmitis. There was great concern that the incidence of bleb-related endo ph-thalmitis would increase significantly with the introduction of antimetabolites as adjuncts for trabe culectomy.^93,94^ However, concerns of an impending epidemic of bleb-related infection seem to have not manifested thus far. In the largest study of bleb-related infection to date, Yamomoto et al.^95^ conducted a prospective, observational cohort study of 1,098 eyes of 1,098 patients who underwent either trabeculectomy or phacotrabeculectomy augmented with MMC and reported a 5-year endophthalmitis incidence of 1.1%. Infection was more commonly associated with bleb leak and younger age in this cohort. A similar incidence was reported by the collaborative initial glaucoma treatment study (CIGTS) group in their long-term review of the 300 patients randomized to the trabeculectomy arm.^96^ In the CIGTS, the 5-year incidence of bleb-related endophthalmitis was 1.1% with no significant increase in endophthalmitis risk in the patients who received adjunctive anti-fibrotics (predominantly 5FU).

Greenfield et al.^94^ retrospectively reviewed the course of 609 eyes of 485 patients who underwent trabeculectomy with MMC and found an endophthalmitis of rate of 2.1%, occurring at a mean time of 19 months after surgery. The incidence of endophthalmitis was significantly greater following an inferior trabeculectomy compared to a superior one. These findings have been corroborated in other studies.^97,98^

Both 5FU and MMC can cause corneal epithelial toxicity. This is due to the anti-proliferative effect on the stem cells in the limbal region, preventing repopulation of corneal epithelium, and resulting in an epithelial defect.^5^ The incidence of epitheliopathy is significantly higher with 5FU, ranging from 17 to 64%, primarily due the repeated localized postoperative injections.^8,99^ This effect is usually limited and self-resolving but may be severe enough to cause persistent, non-healing epithelial defects. Corneal toxicity resulting from MMC can be more serious. Significant cell loss can occur during or immediately after MMC-augmented trabeculectomy.^100^ Inadvertent leakage of MMC at a concentration of 200 μg/ml results in prompt cor neal swelling with marked ultra structural alterations.^101^

### Novel Methods of Antimetabolite delivery

Due to the potential complications and inconsistent results associated with the current method of antimetabolite applicat ion, alter nat ive and more cont rolled methods of d r ug del iver y have be en explored in recent yea rs. However, t hese are still in the experimental phase and as yet, studies have not progressed outside the laboratory.

Various hydrogels have been evaluated in animal models for their ability to provide slow, sustained release of incorporated wound healing modulators to the filtration site and at the same time min imising toxicit y to t he su r rou ndi ng tissues. P(HEMA) hydrogel loaded with MMC has been shown to inhibit conjunctival fibroblast proliferation *in vitro*.^102^ When placed in disks and attached to Ahmed glaucoma valves, it released MMC *in vitro* over 1 to 2 weeks and reduced bleb Inflammation and fibrosis in rabbits.^103^ A bioactive self-assembled peptide loaded with 5FU implanted subconju nctivally adjacent t o t he filt r ation site in r abbit eyes has also demonstrated potential in terms of improving bleb survival and IOP control compared to 5FU alone.^104,105^

The discovery of over-expression of LDL-receptors in activated Tenon's capsule fibroblasts^106^ has led to research on of LDL-chitosan nanoparticles incorporated with MMC as a method of allowing safe and sustained release of MMC following glaucoma surgery.^107^ This approach could potentially allow more targeted delivery of MMC as MMC is confined and protected by the chitosan nanoparticles until the particles bind to the over-expressed LDL receptors on Tenon's fibroblasts and then released. Uptake of the MMC-incorporated LDL-nanoparticles is potentially more vigorous due to the over-expressed LDL receptors. Chitosan nanoparticles are stable in human tissue and are theoretically safe but the safety and efficacy of this new system are yet to be tested in laboratory and clinical trials.

## CONCLUSION

Glaucoma filtration surgery has evolved significantly over the past few decades, and antimetabolites have contributed to better outcomes in terms of postoperative IOP control. Despite advances in surgical technique, the risks associated with the use of antimetabolites in filtration surgery remain very real. Newer techniques in delivering antimetabolites are being investigated as a means of harnessing their anti-fibrotic properties to reliably maintain the efficacy of IOP-lowering yet minimising the risk of complications. Newer agents and materials, including anti-VEGF, are among the exciting developments that may offer a similar level of IOP reduction but without the same level of risk. These will be discussed in Part 2 of this review.

## References

[1] Chen CW (1983). Enhanced int raocular pressure cont rolling ef fective ness of trabeculectomy by local application of mitomycin C.. Trans Asia-Pacific Acad Ophthalmol.

[2] Gressel MG, Parrish RK 2nd, Folberg R (1984). 5-fluorouracil and glaucoma filtering surgery: I. An animal model.. Ophthal mology.

[3] Cordeiro MF, Schultz GS, Ali RR, Bhattacharya SS, Khaw PT (1999). Molecular therapy in ocular wound healing.. Br J Ophthalmol.

[4] Skuta GL, Parrish RK 2nd (1987). Wound healing in glaucoma filtering surgery.. Surv Ophthalmol.

[5] Loon SC, Chew PT (1999). A major review of antimetabolites in glaucoma therapy.. Ophthalmologica.

[6] Crowston JG, Akbar AN, Constable PH, Occleston NL, Daniels JT, Khaw PT (1998). Antimetabolite-induced apoptosis in Tenon's capsule fibroblasts.. Invest Ophthalmol Vis Sci.

[7] Crowston JG, Chang LH, Constable PH, Daniels JT, Akbar AN, Khaw PT (2002). Apoptosis gene expression and death receptor signaling in mitomycin-C-treated human tenon capsule fibroblasts.. Invest Ophthalmol Vis Sci.

[8] Khaw PT, Doyle JW, Sherwood MB, Grierson I, Schultz G, McGorray S (1993). Prolonged localized tissue effects from 5-minute exposures to fluorouracil and mitomycin C.. Arch Ophthalmol.

[9] Nakamura M, Yamamoto M (1994). DNA interstrand crosslinking agents and human ocular fibroblasts: differential sensitivity to mitomycin-C and cis-diaminedichloroplatinum(II).. Exp Eye Res.

[10] Lee DA, Lee TC, Cortes AE, Kitada S (1990). Effect so fmithramycin , mitomycin, daunorubicin and bleomycin on human subcon junctival fibroblast attachment and proliferation.. Invest Ophthalmol Vis Sci.

[11] Skuta GL, Beeson CC, Higginbotham EJ, Lichter PR, Musch DC, Bergstrom TJ, Klein TB, Falck FY Jr (1992). Int raoperative mitomycin versus postoperative 5-fluorouracil in high-risk glaucoma filtering surgery.. Ophthalmology.

[12] Falck FY Jr, Skuta GL, Klein TB (1992). Mitomycin versus 5-fluorouracil antimetabolite therapy for glaucoma filtration surgery.. Semin Ophthalmol.

[13] Palmer SS (1991). Mitomycin as adjunct chemotherapy with trabeculectomy.. Ophthalmology.

[14] Mostafaei A (2011). Augmenting trabeculectomy in glaucoma with subconjunctival mitomycin Cversus subconjunctival 5-fluorouracil: a randomized clinical trial.. Clin Ophthalmol.

[15] El Sayyad F, Belmekki M, Helal M, Khalil M, El-Hamzawey H, Hisham M (2000). Simultaneous subconjunctival and subscleral mitomycin-C application in trabeculectomy.. Ophthalmology.

[16] Prata JA Jr, Minckler DS, Baerveldt G, Lee PP, Heuer DK (1994). Site of mitomycin-C application during trabeculectomy.. J Glaucoma.

[17] Gandolf SA, Vecchi M, Braccio L (1995). Decrease of intraocular pressure after subconjunctival injection of mitomycin in human glaucoma.. Arch Ophthalmol.

[18] Mietz H, Addicks K, Diestelhorst M, Krieglstein GK (1994). Extraocular application of mitomycin C in a rabbit model: cytotoxic effects on the ciliary body and epithelium.. Ophthalmic Surg.

[19] Kee C, Pelzek CD, Kaufman PL (1995). Mitomycin C suppresses aqueous humor fow in cynomolgus monkeys.. Arch Ophthalmol.

[20] Georgopoulos M, Vass C, El Menyawi I, Radda S, Graninger W, Menapace R (2000). Invitro diffusion of mitomycin-C into human sclera after episcleral application: impact of diff usion time.. Exp Eye Res.

[21] Vass C, Georgopoulos M, el Menyawi I, Radda S, Nimmerrichter P (2000). Intrascleral concentration vs depth profile of mitomycin-C after episcleral application: impact of applied concentration and volume of mitomycin-C solution.. Exp Eye Res.

[22] Cheung JC, Wright MM, Murali S, Pederson J E (1997). Inter mediate-term outcome of variable dose mitomycin C filtering surgery.. Ophthalmology.

[23] Lee SJ, Paranhos A, Shields MB (2009). Does titration of mitomycin C as an adjunct to trabeculectomy significantly infuence the intraocular pressure outcome?. Clin Ophthalmol.

[24] Perkins TW, Gangnon R, Ladd W, Kaufman PL, Heatley GA (1998). Trabeculectomy with mitomycin C: intermediate-term results.. J Glaucoma.

[25] Megevand GS, Salmon JF, Scholtz RP, Murray AD (1995). The effect of reducing the exposure time of mitomycin C in glaucoma filtering surgery.. Ophthalmology.

[26] Neelakantan A, Rao BS, Vijaya L, Grandham SB, Krishnan N, Priya VS, Murugeshan R (1994). Effect of the concentration and duration of application of mitomycin C in trabeculectomy.. Ophthalmic Surg.

[27] Kim YY, Sexton RM, Shin DH, Kim C, Ginde SA, Ren J, Lee D, Kupin TH (1998). Outcomes of primary phakic trabeculectomies without versus with 0.5- to 1-minute versus 3- to 5-minute mitomycin C.. Am J Ophthalmol.

[28] Robin AL, Ramakrishnan R, Krishnadas R, Smith SD, Katz JD, Selvaraj S, Skuta GL, Bhatnagar R (1997). A long-term dose-response study of mitomycin in glaucoma filtration surgery.. Arch Ophthalmol.

[29] Vass C, Georgopoulos M, El Menyawi I, Radda S, Nimmer richter P, Menapace R (2000). Intrascleral concentration vs depth profile of mitomycin-C after episcleral application: impact of irrigation.. Exp Eye Res.

[30] Kirwan JF, Lockwood AJ, Shah P, Macleod A, Broadway DC, King AJ, McNaught AI, Agrawal P (2013). Trabeculectomy in the 21st century: a multicenter analysis.. Ophthalmology.

[31] Edmunds B, Thompson JR, Salmon JF, Wormald RP (2001). The National Survey of Trabeculectomy. II. Variations in operative technique and outcome.. Eye (Lond).

[32] Joshi AB, Parrish RK 2nd, Feuer WF (2005). 2002 Survey of the American Glaucoma Society: practice preferences for glaucoma surgery and antifibrotic use.. J Glaucoma.

[33] Reibaldi A, Uva MG, Longo A (2008). Nine-year follow-up of trabeculectomy with or without low-dosage mitomycin-C in primary open-angle glaucoma.. Br J Ophthalmol.

[34] Fontana H, Nouri-Mahdavi K, Lumba J, Ralli M, Caprioli J (2006). Trabeculectomy with mitomycin C: outcomes and risk factors for failure in phakic open-angle glaucoma.. Ophthalmology.

[35] Beckers HJ, Kinders KC, Webers CA (2003). Five-year results of trabeculectomy with mitomycin C.. Graefes Arch Clin Exp Ophthalmol.

[36] Shigeeda T, Tomidokoro A, Chen YN, Shirato S, Araie M (2006). Long-ter m follow-up of in itial trabeculectomy with mitomycin C for primary open-angle glaucoma in Japanese patients.. J Glaucoma.

[37] Mills K B (1981). Trabeculectomy: a retrospective long-ter m follow-up of 444 cases.. Br J Ophthalmol.

[38] Levene RZ (1984). Glaucoma filter ing su rger y: factors that deter mi ne pressure control.. Ophthalmic Surg.

[39] Jacobi PC, Dietlein TS, Krieglstein GK (1998). Adjunctive mito mycin C in primary trabeculectomy in young adults: a long-term study of case-matched young patients.. Graefes Arch Clin Exp Ophthalmol.

[40] Membrey WL, Poinoosawmy DP, Bunce C, Hitchings RA (2000). Glaucoma surgery with or without adjunctive antiproliferatives in nor mal tension glaucoma: 1 int raocula r pressu re cont rol and complications.. Br J Ophthalmol.

[41] Membrey WL, Bunce C, Poinoosawmy DP, Fitzke FW, Hitchings RA (2001). Glaucoma surgery with or without adjunctive antiproliferatives in normal tension glaucoma: 2 Visual field progression.. Br J Ophthalmol.

[42] Mandal AK, Walton DS, John T, Jayagandan A (1997). Mitomycin C-augmented trabeculectomy in refractory congenital glaucoma.. Ophthalmology.

[43] Sidoti PA, Belmonte SJ, Liebmann JM, Ritch R (2000). Trabeculectomy with mitomycin-C in the treatment of pediatric glaucomas.. Ophthalmology.

[44] al-Hazmi A, Zwaan J, Awad A, al-Mesfer S, Mullaney PB, Wheeler DT (1998). Effectiveness and complications of mitomycin C use during pediatric glaucoma surgery.. Ophthalmology.

[45] Mandal AK, Bagga H, Nutheti R, Gothwal VK, Nanda AK (2003). Trabeculectomy with or without mitomycin-C for paediatric glaucoma in aphakia and pseudophakia following congenital cataract surgery.. Eye (Lond).

[46] Azuara-Blanco A, Wilson RP, Spaeth GL, Schmidt CM, Augsburger JJ (1999). Filtration procedures supplemented with mitomycin C in the management of childhood glaucoma.. Br J Ophthalmol.

[47] Beck AD, Wilson WR, Lynch MG, Lynn MJ, Noe R (1998). Trabeculectomy with adjunctive mitomycin C in pediatric glaucoma.. Am J Ophthalmol.

[48] Manners T, Salmon JF, Barron A, Willies C, Murray AD (2001). Trabeculectomy with mitomycin C in the treatment of post-traumatic angle recession glaucoma.. Br J Ophthalmol.

[49] Noble J, Derzko-Dzulynsky L, Rabinovitch T, Birt C (2007). Outcome of trabeculectomy with intraoperative mitomycin C for uveitic glaucoma.. Can J Ophthalmol.

[50] Panek WC, Holland GN, Lee DA, Christensen RE (1990). Glaucoma in patients with uveitis.. Br J Ophthalmol.

[51] Kaburaki T, Koshino T, Kawashima H, Numaga J, Tomidokoro A, Shirato S, Araie M (2009). Initial trabeculectomy with mitomycin C in eyes with uveitic glaucoma with inactive uveitis.. Eye (Lond).

[52] Kiuchi Y, Sugimoto R, Nakae K, Saito Y, Ito S (2006). Trabeculectomy with mitomycin C for treatment of neovascular glaucoma in diabetic patients.. Ophthalmologica.

[53] Takihara Y, Inatani M, Fukushima M, Iwao K, Iwao M, Tanihara H (2009). Trabeculectomy with mitomycin C for neovascular glaucoma: prognostic factors for surgical failure.. Am J Ophthalmol.

[54] Maestrini HA, Cronemberger S, Matoso HD, Reis JR, Márula RV, Filho AD, Sakurai E, Ferreira GA (2011). Late needling of fat filtering blebs with adjunctive mitomycin C: efficacy and safety for the corneal endothelium.. Ophthalmology.

[55] Iwach AG, Delgado MF, Novack GD, Nguyen N, Wong PC (2003). Transconjunctival mitomycin-C in needle revisions of failing filtering blebs.. Ophthalmology.

[56] Mardelli PG, Lederer CM Jr, Murray PL, Pastor SA, Hassanein KM (1996). Slit-lamp needle revision of failed filtering blebs using mitomycin C.. Ophthalmology.

[57] Cheng JW, Cai JP, Li Y, Wei RL (2011). Intraoperative mitomycin C for nonpenetrating glaucoma surgery: a systematic review and meta-analysis.. J Glaucoma.

[58] Alvarado JA, Hollander DA, Juster R P, Lee LC (2008). Ahmed valve implantation with adjunctive mitomycin C and 5-fluorouracil: long-term outcomes.. Am J Ophthalmol.

[59] Costa V P, Azuara-Blanco A, Netland PA, Lesk MR, Arcieri ES (2004). Efficacy and safety of adjunctive mitomycin C during Ahmed Glaucoma Valve implantation: a prospective randomized clinical trial.. Ophthalmology.

[60] Al-Mobarak F, Khan AO (2009). Two-year survival of Ahmed valve implantation in the first 2 years of life with and with out intraoperative mitomycin C.. Ophthalmology.

[61] Abraham LM, Selva D, Casson R, Leibovitch I (2007). The clinical applications of fluorouracil in ophthalmic practice.. Drugs.

[62] Khaw PT, Sherwood MB, MacKay SL, Rossi MJ, Schultz G (1992). Five-minute treatments with fluorouracil, foxuridine and mitomycin have long-term effects on human Tenon's capsule fibroblasts.. Arch Ophthalmol.

[63] Smith S, D'Amore PA, Dreyer EB (1994). Comparative toxicity of mitomycin C and 5-fluorouracil in vitro.. Am J Ophthalmol.

[64] Palanca-Capistrano AM, Hall J, Cantor LB, Morgan L, Hoop J, WuDunn D (2009). Long-term outcomes of intraoperative 5-fluorouracil versus intraoperative mitomycin C in primary trabeculectomy surgery.. Ophthalmology.

[65] Singh K, Mehta K, Shaikh NM, Tsai JC, Moster MR, Budenz DL, Greenfield DS, Chen PP, Cohen JS, Baerveldt GS (2000). Trabeculectomy with intraoperative mitomycin C versus 5-fluorouracil. Prospective randomized clinical trial.. Ophthalmology.

[66] Towler HM, McCluskey P, Shaer B, Lightman S (2000). Long-term follow-up of trabeculectomy with intraoperative 5-fluorouracil for uveitis-related glaucoma.. Ophthalmology.

[67] Heuer DK, Parrish RK 2nd, Gressel MG, Hodapp E, Desjardins DC, Skuta GL, Palmberg PF, Nevérez JA, Rockwood EJ (1986). 5-Fluorouracil and glaucoma filtering surgery. III. Intermediate follow-up of a pilot study.. Ophthalmology.

[68] Ruderman JM, Welch DB, Smith MF, Shoch DE (1987). A randomized study of 5-fuorour acilandfilt ration surgery.. Am J Ophthal mol.

[69] (1996). The Fluorouracil Filtering Surgery Study Group. Five-year follow-up of the Fluorouracil Filtering Surgery Study.. Am J Ophthalmol.

[70] Goldenfield M, Krupin T, Ruderman JM, Wong PC, Rosenberg LF, Ritch R, Liebmann JM, Gieser DK (1994). 5-Fluorou racil ininitial trabeculectomy. A prospective, randomized, multicenter study.. Ophthalmology.

[71] Tsai JC, Feuer WJ, Parrish RK 2nd, Grajewski AL (1995). 5-Fluoro-uracil filtering surgery and neovascular glaucoma. Long-term follow-up of the original pilot study.. Ophthalmology.

[72] Rockwood EJ, Parrish RK 2nd, Heuer DK, Skuta GL, Hodapp E, Palmberg PF, Gressel MG, Feuer W (1987). Glaucoma filtering surgery with 5-Fluorouracil.. Ophthalmology.

[73] Leyland M, Bloom P, Zinicola E, McAlister J, Rassam S, Migdal C (2001). Single intraoperative application of 5-Fluorouracil versus placebo in low-risk trabeculectomy surgery: a randomized t rial.. J Glaucoma.

[74] Rothman RF, Liebmann JM, Ritch R (2000). Low-dose 5-fluorouracil trabeculectomy as initial surgery in uncomplicated glaucoma: long-term follow-up.. Ophthalmology.

[75] Wong TT, Khaw PT, Aung T, Foster PJ, Htoon HM, Oen FT, Gazzard G, Husain R, Devereux JG, Minassian D (2009). The Singapore 5-fluorouracil trabeculectomy study: effects on int raocula r pressu re control and disease progression at 3 years.. Ophthalmology.

[76] Wong MH, Husain R, Ang BC, Gazzard G, Foster PJ, Htoon HM, Wong TT, Oen FT, Khaw PT, Seah SK (2013). The Singapore 5-fuoro uracil trial: intraocular pressure outcomes at 8 years.. Ophthal mology.

[77] WuDunn D, Cantor LB, Palanca-Capistrano AM, Hoop J, Alvi N P, Finley C, Lakhani V, Burnstein A, Knotts SL (2002). A prospective randomized trial comparing intraoperative 5-fluorouracil vs mitomycin C in primary trabeculectomy.. Am J Ophthalmol.

[78] Singh K, Egbert PR, Byrd S (1997). Trabeculectomy with intra-operative 5-fluorouracil vs mitomycin C.. Am J Ophthalmol.

[79] Lamping KA, Belkin JK (1995). 5-fluorouracil and mitomycin C inpseudophakic patients.. Ophthal mology.

[80] Anand N, Khan A (2009). Long-term outcomes of needle revision of trabeculectomy blebs with mitomycin C and 5-fluorouracil: a comparative safety and efficacy report.. J Glaucoma.

[81] Greenfield DS, Liebmann JM, Jee J, Ritch R (1998). Late-onset bleb leaks after glaucoma filtering surgery.. Archives of Ophthalmology.

[82] Lamping KA, Bellows AR, Hutchinson BT, Afran SI (1986). Long-term evaluation of initial filtration surgery.. Ophthalmology.

[83] Katz GJ, Higginbotham EJ, Lichter PR (1995). Mitomycin C vs 5-fuorou racil in high-r isk glaucoma filtering surgery. Extended follow-up.. Ophthalmology.

[84] Ticho U, Ophir A, Storr-Paulsen T, Norregaard JC, Ahmed S, Storr-Paulsen A Late complications after glaucoma filtering surgery with adjunctive 5-fluorouracil.. Am J Ophthalmol 1993100.

[85] Dhingra S, Khaw PT (2009). The moorfields safer surgery system.. Middle East Afr J Ophthalmol.

[86] Wells AP, Cordeiro MF, Bunce C, Khaw PT (2003). Cystic bleb formation and related complications in limbus vs fornix-based conjunctival faps in pediatric and young adult trabeculectomy with mitomycin C.. Ophthal mology.

[87] Cordeiro MF, Constable PH, Alexander RA, Bhattacharya SS, Khaw PT (1997). Effect of varying the mitomycin C treatment area in glaucoma filtration surgery in the rabbit.. Investigative Ophthalmol and Visual Science.

[88] Kupin TH, Juzych MS, Shin DH, Khatana AK, Olivier MM (1995). Adjunctive mitomycin C in primary trabeculectomy in phakic eyes.. Am J Ophthalmol.

[89] Zacharia PT, Deppermann SR, Schuman JS (1993). Ocular hypotony after trabeculectomy with mitomycin C.. Am J Ophthal mol.

[90] Costa VP, Wilson RP, Moster MR, Schmidt CM, Gandham S (1993). Hypotony maculopathy following the use of topical mitomycin C in glaucoma filtration surgery.. Ophthalmic Surgery.

[91] Stamper RL, McMenemy MG, Lieberman MF (1992). Hypotonous maculopathy after trabeculectomy with subconjunctival 5-fluorouracil.. Am J Ophthalmol.

[92] Seah SK, Prata JA Jr, Minckler DS, Baerveldt G, Lee PP, Heuer DK (1995). Hypotony following trabeculectomy.. J Glaucoma.

[93] Katz LJ, Cantor LB, Spaeth GL (1985). Complications of surgery in glaucoma. Early and late bacterial endophthalmitis following glaucoma filtering surgery.. Ophthalmology.

[94] Greenfield DS, Suner IJ, Miller MP, Kangas TA, Palmberg PF, Flynn HW Jr (1996). Endophthalmitis after filtering surgery with mitomycin.. Archives of Ophthalmology.

[95] Yamamoto T, Sawada A, Mayama C (2014). The 5-year incidence of bleb-related infection and its risk factors after filtering surgeries with adjunctive mitomycin C: collaborative bleb-related infection incidence and treatment study 2.. Ophthalmology.

[96] Zahid S, Musch DC, Niziol LM, Lichter PR (2013). Risk of endophthalmitis and other long-term complications of trabeculectomy in the collaborative initial glaucoma t reatment study (CIGTS).. Am J Ophthalmol.

[97] Higginbotham EJ, Stevens RK, Musch DC (1996). Bleb-related endophthalmitis after trabeculectomy with mitomycin C.. Ophthalmology.

[98] Song A, Scott IU, Flynn HW Jr, Budenz DL (2002). Delayed-onset bleb-associated endophthalmitis: clinical features and visual acuity outcomes.. Ophthalmology.

[99] (1989). The fluorouracil filtering surgery study group. Fluorouracil filtering surgery study one-year follow-up.. Am J Ophthalmol.

[100] Storr-Paulsen T, Norregaard JC, Ahmed S, Storr-Paulsen A (2008). Corneal endothelial cell loss after mitomycin C-augmented trabeculectomy.. J Glaucoma.

[101] McDermott ML, Wang J, Shin DH (1994). Mitomycin and the human corneal endothelium.. Archives of Ophthalmology.

[102] Blake DA, Sahiner N, John VT (2006). Inhibition of cell proliferation by mitomycin C incorporated into P(HEMA) hydrogels. J Glaucoma.

[103] Sahiner N, Kravitz DJ, Qadir R (2009). Creation of a drug-coated glaucoma drainage device using polymer technology:in vitro and in vivo studies.. Archives of Ophthalmology.

[104] Liang L, Xu XD, Zhang XZ, Feng M, Peng C, Jiang FG (2010). Prevention of filtering surgery failure by subconjunctival injection of a novel peptide hydrogel into rabbit eyes.. Biomedical Materials.

[105] Xu XD, Liang L, Chen CS (2010). Peptide hydrogel as an int raocular drug delivery system for in hibition of postoperative scarring formation.. ACS applied materials and interfaces.

[106] Chen HY, Ge J, Guo Y, Jin CJ, Lan YQ, Lin MK (2003). The inhibition effect of photodynamic on human Tenon capsule fibroblast cells.. (Zhonghua yan ke za zhi) Chi nese J Opht hal mology.

[107] Shao T, Li X, Ge J (2011). Target drug delivery system as a new scar ring modulation after glaucoma filtration surgery.. Diagnostic Pathology.

